# Case Report: The first Korean familial case of *BCAP31*-related deafness, dystonia, and cerebral hypomyelination

**DOI:** 10.3389/fped.2024.1488095

**Published:** 2025-01-22

**Authors:** Yoong-A Suh, Jisun Hwang, Go Hun Seo, Rin Khang, Jang Hoon Lee, Moon Sung Park, Young Bae Sohn

**Affiliations:** ^1^ Department of Pediatrics, Ajou University Hospital, Ajou University School of Medicine, Suwon, Republic of Korea; ^2^ Department of Radiology, Ajou University Hospital, Ajou University School of Medicine, Suwon, Republic of Korea; ^3^ Department of Medical Genetics, 3billion, Inc., Seoul, Republic of Korea; ^4^ Department of Medical Genetics, Ajou University Hospital, Ajou University School of Medicine, Suwon, Republic of Korea

**Keywords:** DDCH syndrome, *BCAP31*, whole genome sequencing, x-linked intellectual disability, sensorineural hearing loss

## Abstract

Deafness, dystonia, and central hypomyelination (DDCH) syndrome (OMIM #300475) is a rare X-linked genetic disorder characterized by developmental delays, deafness, central hypomyelination, and dystonia. We report the first Korean familial case involving twin boy and girl carrying a novel pathogenic *BCAP31* variant which was inherited from their mother. The male proband, born prematurely with very low birth weight (VLBW), exhibited severe global developmental delay, microcephaly, failure to thrive, dystonia, seizures, sensorineural hearing loss (SNHL) requiring cochlear implantation, and mild facial dysmorphism. A brain MRI revealed white matter atrophy, thinning of the corpus callosum, and delayed myelination. The twin sister presented with mild developmental delays and bilateral SNHL but did not experience seizures or dystonia. Their mother also had bilateral SNHL. Whole genome sequencing identified a hemizygous pathogenic variant, c.247C>T (p.Gln83Ter), in the *BCAP31* in the proband. The variant was also found in his mother and twin sister, who exhibited less severe symptoms. Early genetic evaluation via next-generation sequencing is crucial for timely diagnosis and intervention, particularly in VLBW infants with genetic disorders. This report expands the understanding of genotype-phenotype correlations in DDCH syndrome and highlights the variable phenotypes in manifesting females.

## Introduction

Deafness, dystonia, and central hypomyelination (DDCH) syndrome (OMIM #300475) is a rare X-linked genetic disorder characterized by developmental delays, deafness, central hypomyelination, and various other neurological symptoms. In 2013, pathogenic variants resulting in loss of function in *BCAP31* were discovered in seven unrelated males with severe developmental delay, sensorineural hearing loss (SNHL), and dystonia (1). The *BCAP31* located on the human chromosome Xq28 (2) encodes B-cell receptor-associated protein 31 (BCAP31), a transmembrane protein, primarily located in the endoplasmic reticulum that plays a role in protein trafficking and apoptosis regulation. Because BCAP31 plays a crucial role in endoplasmic reticulum function, it is expressed in various tissues, including the central nervous system (2–4). *BCAP31* is flanked by two genes: *SLC6A8* on its centromeric side and *ABCD1* on its telomeric side. Individuals who have deletions encompassing *SLC6A8*, *BCAP31*, and *ABCD1* are known to be associated with dystonia, profound intellectual disability, liver dysfunction, SNHL, and early death that are more severe than the symptoms observed in patients with isolated intragenic *BCAP31* mutations (1, 5–8).

Since 2013, 29 male patients with hemizygous loss-of-function mutations in *BCAP31* have been reported (1, 9–13). As DDCH is inherited in an X-linked manner, most reported patients are males. However, there are a few females with heterozygous *BCAP31* mutations who manifest symptoms due to skewed X inactivation (13, 14). Here, we report the first familial case of DDCH syndrome in a Korean boy and his twin sister carrying a pathogenic variant in *BCAP31*, inherited from a mother with milder symptoms.

## Case description

The proband, a 2-year-10-month-old boy, was born at 30 weeks and 1 day of gestation via emergency cesarean section due to fetal distress. He was the first dizygotic twin born to nonconsanguineous Korean parents. At birth, his height was 38 cm (Z-score, −0.48), weight was 1240 g (Z-score, −0.49), and head circumference was 25.5 cm (Z-score, −1.40). The second twin was a girl, born weighing 1180 g (Z-score, −0.48), with a height of 37 cm (Z-score, −0.64), and a head circumference of 26 cm (Z-score, −0.75). Their mother was 35 years old, had bilateral SNHL, and had been wearing hearing aids since the age of seven. She also had diabetes, which was well-controlled with insulin, and was taking methimazole for hyperthyroidism. Her intelligence level was normal. Her parents and siblings (2 sisters) had no specific medial history including hearing loss or neurologic disorders.

The twins were admitted to the neonatal intensive care unit (NICU) for prematurity care. Initially, both developed respiratory distress syndrome, which was treated with surfactant therapy. No distinct facial dysmorphism or other issues were found during the physical examination.

During hospitalization, brain ultrasonography was performed. Grade I germinal matrix hemorrhage was observed in the proband, whereas the twin sister's results were normal. After they were able to regulate their body temperature and demonstrated good sucking ability, they were discharged from the NICU at a corrected age of 36 weeks.

The proband showed abnormal bilateral findings during newborn screening for hearing loss using an automated auditory brainstem response (AABR). Follow-up brainstem audiometry conducted at four months (two months of corrected age) revealed bilateral SNHL. He wore bilateral hearing aids and underwent surgery for a right cochlear implant at the age of one year and six months, with plans for the left side one year later.

**Figure 1:**
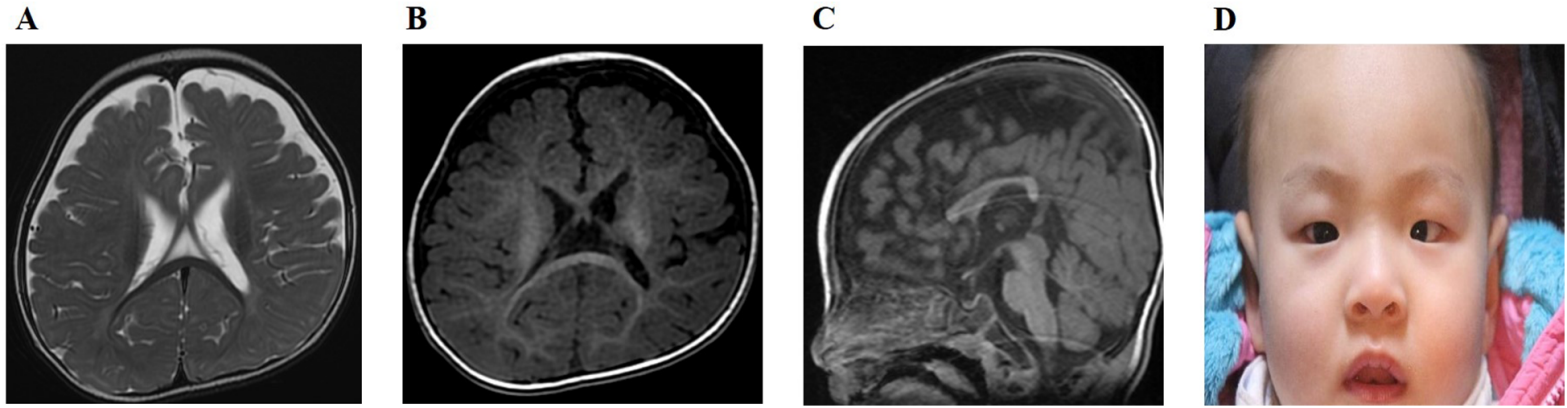
**(A)** Axial T2-weighted image of the proband at 10 months shows marked brain atrophy, particularly in the white matter. **(B)** Axial T1-weighted image of the proband at 10 months displays delayed myelination. **(C)** Sagittal T1-weighted image of the proband at 10 months demonstrates thinning of the corpus callosum. **(D)** At the follow-up examination at two years of age, the proband exhibited mild facial dysmorphism characterized by downward slanting palpebral fissures, hypertelorism, esotropia, and a tented upper lip vermilion.

At seven months (five months of corrected age), developmental delays in both cognitive and motor functions were noted, prompting rehabilitation therapy. He was unable to control his head or trunk, and no significant eye contact was observed. His mental developmental index (MDI) score was 55, and his psychomotor developmental index (PDI) score was 57, as tested using the Bayley Scale of Infant Development. At that time, he experienced two episodes of seizures, with the third occurring at 10 months. Electroencephalography revealed slow and disorganized rhythms in both hemispheres, which led to the initiation of anticonvulsant medication. Brain MRI revealed marked brain atrophy, especially in the white matter, thinning of the corpus callosum, and delayed myelination (Figures 1A–C). At the age of two years, involuntary movements of the arms and trunk were noted, and he was unable to control his trunk or stand on his own. He could not speak a word and only produced babbling sounds. Bilateral esotropia was also observed. His growth velocity worsened, and he had failure to thrive in both height (80 cm; Z-score, −2.09) and weight (8.3 kg, Z-score, −3.55). He also had microcephaly (42.7 cm, Z-score, −3.05). The follow-up brain MRI conducted at two years was limited due to artifacts caused by the right cochlear implant; however, brain atrophy and delayed myelination were still observed.

The proband's twin sister also showed abnormal AABR results during NICU hospitalization and was diagnosed with bilateral SNHL. She had been receiving hearing aids in both ears since the age of one. In contrast to the proband, she exhibited mild developmental delays in cognition and motor skills. She began walking on her own at 18 months of age, and her receptive language skills were in the normal range; however, mild delays were noted in expressive language at two years of age. She underwent speech therapy. No other neurological symptoms, such as seizures or dystonia, were observed. Her growth status was also better than that of the proband: she had a height of 82 cm (Z-score, −0.98), weight of 9.3 kg (Z-score, −1.39), and head circumference of 45 cm (Z-score, −1.62) at the age of two.

**Figure 2:**
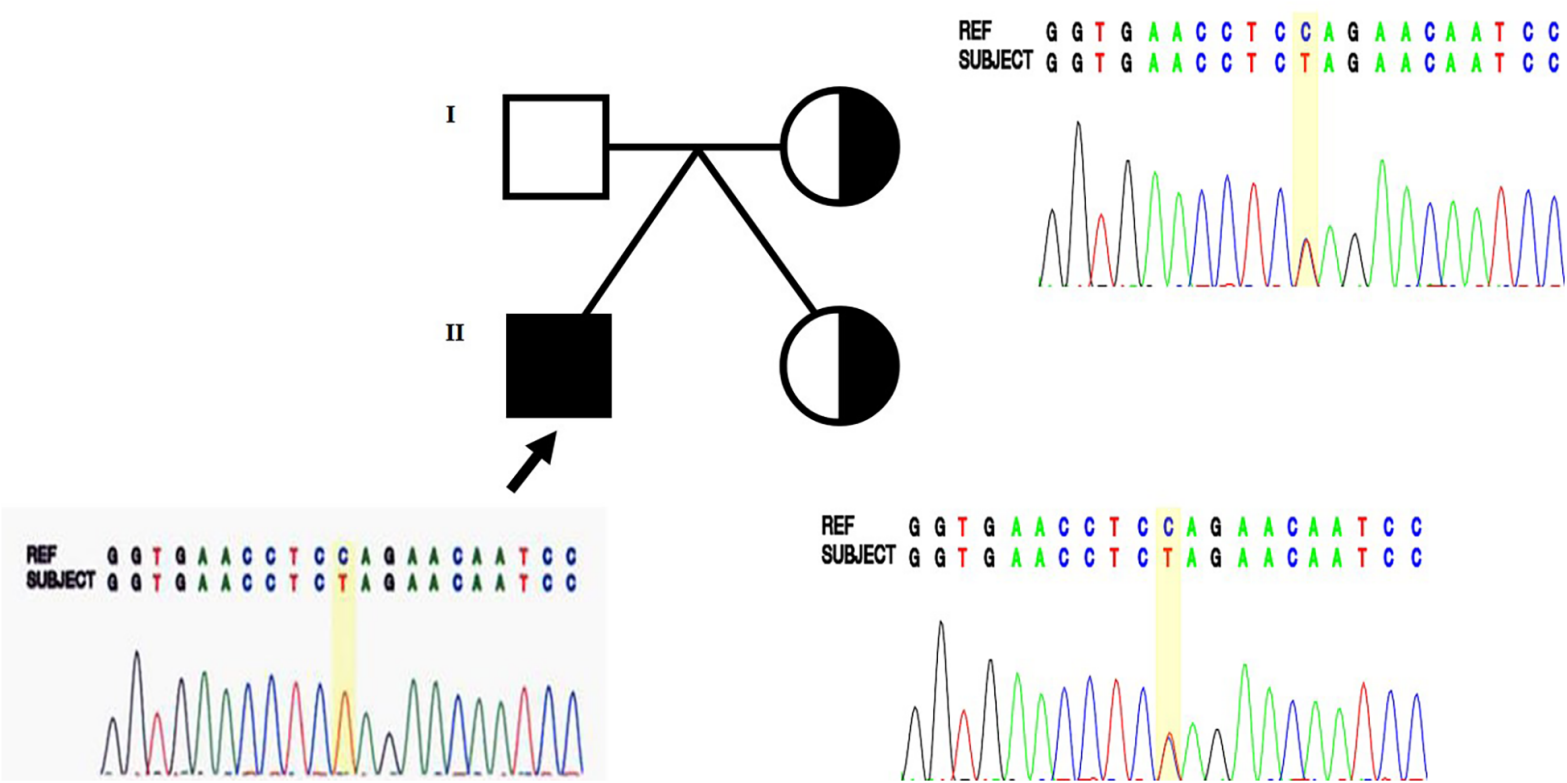
Sanger sequencing identified the hemizygous pathogenic variant (c.247C>T, p.Gln83Ter) in *BCAP31* in the male proband (arrow), and the variant was also found heterozygously in the mother and twin sister, who had a milder phenotype.

The proband was referred to the genetics clinic for assessment of the underlying genetic causes for his global developmental delay (GDD), bilateral SNHL, seizures, and dystonia at two years of age. Karyotyping and microarray analyses were normal. Whole genome sequencing (WGS) was used to identify the monogenic causes. WGS and subsequent Sanger sequencing identified a hemizygous pathogenic variant in *BCAP31*: c.247C>T (p.Gln83Ter) (Figure 2). Familial segregation analysis using Sanger sequencing was performed on the mother and twin sister. The same heterozygous pathogenic variant was found in both the mother and the twin sister.

After the genetic diagnosis was made, a careful follow-up physical examination of the proband showed mild facial dysmorphism, including a downward-slanting palpebral fissure, esotropia, hypertelorism, and tented upper lip vermilion (Figure 1D), which were not remarkable when he was hospitalized in the NICU for prematurity.

At the last follow-up examination, the proband was two years and 10 months old. He was still unable to stand and was undergoing physical therapy. Owing to his hyperkinetic movements, he had difficulty maintaining balance. He could only babble and did not yet understand words. He is currently seizure-free and is taking anticonvulsants. He is undergoing rehabilitation and regularly visits multiple specialists including gastroenterology, neurology, genetics, otolaryngology, and ophthalmology. On the other hand, at same times, the twin sister was able to walk on her own and had no significant problems with either gross or fine motor skills. She is currently undergoing speech therapy, can speak more than 10 words, and attends a daycare center.

## Discussion

Herein, we describe, for the first time, Korean familial cases of DDCH syndrome with a novel pathogenic variant in *BCAP31*. The proband had severe GDD, microcephaly, failure to thrive, generalized dystonia, seizures, bilateral SNHL requiring cochlear implantation, and mild facial dysmorphism. Prominent white matter atrophy, thinning of the corpus callosum, and hypomyelination were detected on the brain MRI. These clinical features were similar to those previously described in male patients. Patients with intragenic *BCAP31* mutations often show permanent or intermittent liver enzyme elevations. Our proband also exhibited elevated liver enzyme levels during illness. The clinical features and diagnostic findings, compared with those of previous cases, are summarized in Table 1. WGS identified a hemizygous nonsense variant (c.247C>T, p.Gln83Ter) in *BCAP31* in our proband, which was predicted to result in a loss of function. Whalen et al. (13) demonstrated that the phenotype in males with intragenic loss of function variants was more severe than that in males with missense variants. Our proband exhibited failure to thrive and severe GDD with various neurological manifestations.

**Table 1:** Comparison of phenotype and genotype of affected individuals.

	Cacciagli et al. (1)							Vittal et al. (9)		Albanyan et al. (10)	Rinaldi et al. (11)	Shimizu et al. (12)	Kao et al. (14)	Whalen et al. (13)	Current patients	
	Family 1		Family 2				Family 3	Family 1		Family 1	Family 1	Family 1	Family 1	14 Families	Family 1	
	*n* = 2		*n* = 4				*n* = 1	*n* = 2		*n* = 1	*n* = 1	*n* = 1	*n* = 1	*n* = 18	*n* = 2	
*BCAP31* variant	c.192-2A>G (p.Ile64fs*25)		Exon 8 deletion				c.97C>T (p.Gln 33*)	c.261_266del (p.Leu87_ Leu89delinsPhe)		c.533_536 dup (p.Ser180 Alafs*6)	c.709_721 del (p.Val237 Trpfs*69)	c.97C>T (p.Gln33*)	c.92G>A (p.Arg31Lys)	Variable nonsense (13/18), missense (5/18)	c.247C>T (p.Gln83Ter)	
Inheritance	mat	mat	mat	mat	mat	mat	mat	mat	mat	mat	*de novo*	mat	*de novo*	16 mat, 1 *de novo*, 1 nd	mat	mat
Gender	male	male	male	male	male	male	male	male	male	male	male	male	female	16 males, 2 females	male	female
Mother's phenotype	NA		NA				NA	NA		SNHL	NA	NA	NA	3 SNHL, 1 mild ID, 2 LOS, 1 nystagmus, 7 asymptomatic	SNHL	
Age of last exam	22 year	11 year	NA	10 months	12 months	13 year	3 year	21 year	NA	4.5 year	3 year	8.8 year	5 year	2.3––32 year	2.8 year	2.8 year
Age of onset	Congenital	Congenital	Congenital	6 weeks	6 weeks	Congenital	Congenital	2m	NA	First week	Congenital	6 months	6 months	NA	Congenital	1 year
Age of death	24 year	13 year	7 months	1 year	2 year	Living	3 year	Living	Living	Living	Living	Living	Living	NA	Living	Living
Deafness	+	+	NA	+	+	+	+	+/-	+	+	+	+	+	13/16, 1/2 +	+	+
Dystonia	+	+	NA	+	+	+	+	+	+	+	+	+	+	16/16, 0/2 +	+	−
Seizure	+	+	NA	−	−	+	−	−	−	−	+	−	+	4/16, 1/2 +	+	−
Microcephaly	+	+	NA	NA	NA	+	+	NA	NA	+	+/−	+	NA	9/16, 1/2 +	+	+
Cognitive development	none	none	none	none	none	Simple sign language and scribbling	none	Basic academic skills, some language, reading	none	Severe DD	NA	No speech	profound DD	12/16: absent language, 1/2: delayed dysarthria	severe DD	Expressive language delay
Motor development	none	none	none	none	Head control (1 year)	Head control (6 month) Sitting(5 year) Wheelchair (8 year)	Head control (1 year)	cannot walk	cannot walk	Unable to control head movement	Wheelchair-bound with incomplete head control l (2 year and 4 months)	Head control (2 year and 6 months)	profound DD	2/2 females can walk, 3/16 males can sit, 1/16 males can walk(ataxia)	Head control (1 year) Scooting (1 year and 8 months)	Walking (1 year 6 months)
Elevated liver enzyme	NA	NA	NA	NA	NA	NA	NA	−	−	+	+	Cholecystitis with gallbladder stones	+	10/16, 0/2	+	−
Failure to thrive	+	+	NA	+	+	+	+	NA	NA	+	+	+	NA	NA	+	−
Ocular	Congenital strabismus	Congenital strabismus, abnormal eye movement	NA	Optic atrophy	Congenital strabismus	Congenital strabismus, optic atrophy	Congenital strabismus	NA	NA	NA	Nystagmus, bluish sclerae, inconstant esotropia	Right eye esotropia	NA	NA	Congenital strabismus	Left eye esotropia
Brain MRI	NA	Periventricular hypomyelination, atrophy of cerebral cortex, cerebellum	NA	NA	NA	Periventricular hypomyelination	Diffuse hypomyelination, atrophy of CC, frontal lobe, white matter	Delayed myelination, atrophy in posterior occipital lobes, hypoplasia of superior cerebellar vermis, thinning of CC	NA	Bilateral globus pallidus hyperintensity	Periventricular hypomyelination	Thalamus and globus pallidus hyperintensity, cerebral atrophy	Delayed myelination, mild cerebral atrophy with bilateral ventricular dilatation, thinning of CC	Cortical atrophy(4/18) abN BG(2/18) vermis hypoplasia(2/18) thin corpus callosum (4/18)	Brain atrophy, thinning of CC, delayed myelination	NA

mat, maternal; NA, not available; nd, not determined; y, years; m, months; +, have phenotype; −, characterless; SNHL, sensorineural hearing loss; ID, intellectual disability; LOS, late onset seizure.

^a^ Genotypes identified in Whalen et al.; DD, developmental delay; abN, abnormal; CC, corpus callosum.

In previous studies, the timing of brain MRI varied widely, ranging from three months to adulthood (1, 10, 12, 13). Owing to the lack of serial MRI scans in previous cases, it was unclear exactly when hypomyelination or cortical atrophy first occurred. Follow-up brain imaging assessments are necessary to demonstrate the evolution of brain hypomyelination in the proband (12). Furthermore, continued multidisciplinary clinical follow-up is necessary to monitor various symptoms.

The current report includes not only a male patient with a typical phenotype but also familial female cases (mother and twin sister) with attenuated clinical manifestations. There are a few manifesting female cases reported in previous studies (13, 14). Whalen et al. (13) reported two female cases: one had drop attack seizures and mild intellectual disability, and the other had severe developmental delay, was able to walk by four years of age, and had hearing loss. Kao et al. (14) reported a female patient with symptoms similar to those of our proband, including deafness, seizures, developmental delay, and dyskinetic movements. This indicates that the severity spectrum in females may be wide. In mild cases, symptoms may not appear initially, leading to a higher likelihood of later diagnosis than in severe cases. In fact, the first case described by Whalen et al. (13), the diagnosis was made after the patient experienced seizures at the age of 17 years. By contrast, female patients with severe symptoms were diagnosed between the ages of three and five (13, 14). In addition, reports of symptomatic mothers have also been documented (10, 13). Their symptoms were reported to be mild, including hearing loss, mild intellectual disability, or learning disabilities without severe manifestations (10, 13). Although the twin sister in our familial case has not yet experienced seizures, neurological symptoms may develop in the future. Therefore, she requires continuous follow-up monitoring. Heterozygous female carriers of X-linked genetic diseases are generally unaffected because X chromosome inactivation involves random selection to silence one of the X chromosomes early in mammalian female development (15, 16). However, female carriers may develop symptoms if they have skewed X-chromosome inactivation (XCI), in which the X chromosome carrying the wild-type allele is preferentially inactivated (14, 16). In symptomatic females, the severity of phenotypes may vary depending on the skewed XCl ratio. In our study, the inability to compare and analyze the XCl ratios was a limitation.

There has been only one reported case of DDCH syndrome in a premature boy who exhibited chaotic motor patterns from birth, episodic myoclonic movements, and required gastrostomy tube insertion due to feeding difficulties (11). In our case, the proband was born as a very low birth weight infant (VLBWI), and the only notable sign of the disease was abnormal AABR results during hospitalization in the NICU. Other clinical manifestations, even facial dysmorphisms, were also difficult to identify. In cases of premature VLBWI, it is difficult to suspect a specific syndromic disorder with mild dysmorphism because symptoms may not appear due to prematurity (17, 18). Therefore, early genetic evaluation using next-generation sequencing (NGS)-based techniques and genotype-first approaches may be useful for early diagnosis and intervention to improve the outcomes of VLBWI with genetic disorders. Many recent studies have quickly identified genetic disorders using NGS-based tests, including rapid targeted gene panel sequencing, exome sequencing, and genome sequencing, in critically ill newborns or pediatric patients before their clinical phenotypes are evident (17–21). Zhu et al. (22) reported the usefulness of genomic screening, in combination with hearing screening, to detect genetic hearing loss in newborns in the NICU. Although we were not able to apply these early diagnostic approaches for genetic disorders during the NICU care of our proband, WGS performed at two years of age enabled the exact molecular diagnosis of DDCH syndrome, which is difficult to diagnose clinically because of its ultra-rarity and variable neurological symptoms.

In conclusion, we report the first Korean familial case of DDCH syndrome. This report could contribute to expand the understanding of genotype and phenotype of DDCH syndrome by introducing a novel pathogenic nonsense variant in *BCAP31* (c.274C>T, p.Gln83Ter) and the phenotype by demonstrating its association with both severe and mild phenotypes in male and female carriers, respectively.

## Data Availability

The datasets presented in current study are available in the ClinVar (SCV005199871).
